# Correlation Between Serum Ferritin and Degree of Hepatic Fibrosis on Fibroscan in Thalassemic Patients

**DOI:** 10.7759/cureus.42069

**Published:** 2023-07-18

**Authors:** Muhammad Shujat Ali, Munira Borhany, Aqsa Javed Butt, Rabeea Munawar Ali, Syed Kashif, Muhammad Wahaj, Tahir Shamsi

**Affiliations:** 1 Clinical Hematology, National Institute of Blood Diseases and Bone Marrow Transplantation, Karachi, PAK; 2 Internal Medicine, Dr. Ruth K. M. Pfau Civil Hospital Karachi, Karachi, PAK; 3 Medicine, Peshawar Institute of Medical Sciences, Peshawar, PAK

**Keywords:** hcv, crp, fibrosis, ferritin, thalassemia

## Abstract

Aim and objective

This study aimed to examine the relationship between serum ferritin levels and the degree of hepatic fibrosis as detected on Fibroscan in thalassemia patients.

Materials and methods

This was a single-center and cross-sectional study conducted from April 2021 to December 2022. The sample population comprised 55 beta-thalassemia patients receiving treatment at the National Institute of Blood Diseases and Bone Marrow Transplantation, Karachi, Pakistan. The data was compiled through a series of patient interviews, an examination of medical records and was analyzed to obtain the results. Descriptive statistics were used for several variables, including diagnosis, Fibroscan score, blood group, comorbidity, visceromegaly, consanguinity, serum glutamate pyruvate transaminase (SGPT), viral markers, and C reactive protein (CRP). The correlation analysis was done using Spearman’s correlation test.

Results

There were 55 participants in the study, 40 of whom were male and 15 of whom were female. The mean age of the patients was eight years, while the average age at diagnosis was nine months with a transfusion frequency of every 20 days. Spearman's rho (r = 0.287), and the significant value of (p = 0.033) confirmed a statistically significant positive correlation between serum ferritin levels and hepatic fibrosis. On Fibroscan, 74.5% of patients had F0-F1 stage fibrosis followed by 14.5% of the patients having F2 stage fibrosis. HCV seropositivity was the most prevalent comorbidity among the patients. 80% of patients had serum ferritin levels greater than 1000 ug/mL. Hepatosplenomegaly was present in 43.6% of the patients. 78.2% of patients were born out of consanguineous marriages.

Conclusion

In conclusion, this study found a statistically significant positive correlation between serum ferritin levels and hepatic fibrosis in beta-thalassemia patients. The study emphasizes the significance of monitoring serum ferritin levels in thalassemia patients to prevent hepatic fibrosis.

## Introduction

Beta-thalassemia is an inherited blood disorder that impacts the production of hemoglobin, the protein responsible for oxygen transport within red blood cells [1]. Mutations in the beta-globin gene cause decreased or absent production of the beta-globin chain of hemoglobin, resulting in chronic hemolytic anemia and various complications [2]. The global burden of beta-thalassemia carriers has been reported to be 80 million and each year 23000 babies are born with beta-thalassemia major alone [3]. In Pakistan, it has been reported that there are more than 10 million carriers of beta-thalassemia trait and each year, around 5000 new cases of beta-thalassemia major are reported [4].

Iron overload is one of the most prevalent complications of beta-thalassemia, caused by repeated blood transfusions and excessive assimilation of dietary iron [5]. This iron excess can induce oxidative stress and inflammation in the liver, heart, and endocrine tissues. Due to its function in modulating iron metabolism and high iron content, the liver is particularly susceptible to iron excess [6]. Iron excess in the liver can cause hepatic fibrosis, a condition characterized by an excessive buildup of extracellular matrix proteins, which can eventually lead to cirrhosis and hepatic failure [7]. Up to fifty percent of patients with beta-thalassemia develop hepatic fibrosis [5]. Variability exists in the severity and progression of hepatic fibrosis in beta-thalassemia; the underlying mechanisms are incompletely understood [8].

Serum ferritin levels have been suggested as an indicator of iron excess in beta-thalassemia, as they reflect the quantity of iron deposited in the body. As such serum ferritin levels have been shown to correlate with liver iron concentration, a major risk factor for hepatic fibrosis and cirrhosis [9]. The clinical significance of serum ferritin levels as a marker of hepatic fibrosis in beta-thalassemia remains ambiguous.

According to Parakh et al. [10], Fibroscan is a non-invasive imaging technique that assesses hepatic rigidity as a marker of fibrosis and is a reliable and accurate method for assessing hepatic fibrosis in beta-thalassemia.

This study aims to examine the relationship between serum ferritin levels and fibrosis on Fibroscan in beta-thalassemia patients. Understanding the relationship between serum ferritin levels and hepatic fibrosis on Fibroscan may provide valuable insights into the pathophysiology of hepatic fibrosis in beta-thalassemia. It may contribute to an improvement in the diagnosis and treatment of this complication.

## Materials and methods

Study design

This was a single-center, cross-sectional study conducted from April 2021 to December 2022.

Study population

The sample population comprised 55 beta-thalassemia patients receiving treatment at the National Institute of Blood Diseases and Bone Marrow Transplantation, Karachi. Patients of any age and gender who fulfilled the inclusion criteria were included in the study. The inclusion criteria were: confirmed beta-thalassemia diagnosis by hemoglobin electrophoresis and/or genetic analysis, ferritin, and Fibroscan results. Following patients were excluded from the study: any evidence of active infection and history of bone marrow transplant.

Data collection

The data was compiled through a series of patient interviews and an examination of medical records. The information gathered for each patient was: demographic information (age and gender), clinical information (beta-thalassemia type, age at diagnosis, frequency of transfusions, age at first transfusion, comorbidities and consanguineous marriage in parents), laboratory results (serum ferritin levels, complete blood counts, blood group, serum glutamate pyruvate transaminase (SGPT), creatinine, CRP) and Fibroscan results.

All laboratory information was extracted from the medical records of the patients. Results of the Fibroscan were obtained during routine patient consultations.

Data analysis

The data were analyzed using SPSS (IBM Corp. Released 2019. IBM SPSS Statistics for Windows, Version 26.0. Armonk, NY: IBM Corp). The data were summarized using descriptive statistics, including means, frequencies, and percentages. Spearman's correlation coefficient was used to analyze correlations between serum ferritin levels and Fibroscan results. A p-value of 0.05 or less was deemed statistically significant.

Ethical considerations

This study was approved by the Ethics Committee of the National Institute of Blood Diseases and Bone Marrow Transplantation, Karachi (IRB #: NIBD/IRB-214/07-2021). Prior to participating in the study, all patients provided written informed consent. The confidentiality of patient data was maintained throughout the study.

## Results

Of the total 55 patients, 72.7% were male, 27.3% were female, and 94.5% were diagnosed with beta-thalassemia major. Only 5.5% of the patients had beta-thalassemia intermedia. The average age of the patients was eight years, ranging between two and 25 years. The mean age at diagnosis was 9.45 months, ranging from three to 24 months, and the mean age at first transfusion was 8.48 months, also ranging from three to 24 months. On average, transfusions occurred every 20 days, with a minimum of 15 days and a maximum of 30 days. The mean creatinine level was 0.38, ranging between 0.20 and 0.70. The mean CRP level was 1.09, with values ranging from 1 to 2 (Table 1).

**Table 1:** Mean values for Age, Transfusion, Creatinine, and CRP levels CRP: C reactive protein

Variables	Mean	Minimum	Maximum
Age	7.9455	2.00	25.00
Age at Diagnosis in Months	9.4545	3.00	24.00
Age at First Transfusion in Months	8.4828	3.00	24.00
Frequency of Transfusion in Days	20.0000	15.00	30.00
Creatinine	0.3818	0.20	0.70
CRP	1.0909	1.00	2.00

The results showed that the majority of patients (80%) had high serum ferritin levels (>1000 ng/mL), followed by mildly elevated levels (337-1000 ng/ml) in 12.7% and normal levels (24-336 ng/ml) in 7.3% of patients. According to the Fibroscan results, 74.5% of the patients had fibrosis at the F0-F1 stage, 14.5% at the F2 stage, 9.1% at the F3 stage, and only 1.8% had fibrosis at the F4 stage. In terms of blood group, B+ (29.1%) was the most prevalent, followed by A+ (23.6%) and O+ (21.1%). Only 14.5% of the patients had comorbidities such as pure white cell aplasia, autoimmune hemolytic anemia, Gaucher disease, and hepatitis C while 85.5% had no comorbidities. Hepatosplenomegaly was present in 43.6% of the patients. Only 21.8% of the patients were not born from consanguinous marriages, while 78.2% were. Only 21.8% of the patients had elevated levels of serum glutamate pyruvate transaminase (SGPT), while 78.2% had normal levels. About 7.3% of the patients tested positive for hepatitis C virus (HCV) by antibody screening test which was confirmed by polymerase chain reaction viral load, while 92.7% were negative for hepatitis B virus (HBV) and HCV by antibody screening test. All patients had normal CRP levels, which indicated the absence of any active infection (Table 2). 

**Table 2:** Frequencies and Percentages of the variables BTM: beta thalassemia major; BTI: beta thalassemia intermedia; NKCM: no known co-morbids; HCV: hepatitis C virus; AIHA: autoimmune hemolytic anemia; SGPT: serum glutamate pyruvate transaminase; HBV: hepatitis B virus; DCT: direct coomb's test.

		Frequency	Percent
GENDER	Male	40	72.7
	Female	15	27.3
DIAGNOSIS	BTM	52	94.5
	BTI	3	5.5
FIBROSCAN	F0-F1	41	74.5
	F2	8	14.5
	F3	5	9.1
	F4	1	1.8
BLOOD GROUP	A+	13	23.6
	A-	4	7.3
	B+	16	29.1
	B-	2	3.6
	AB+	5	9.1
	AB-	1	1.8
	O+	12	21.8
	O-	2	3.6
COMORBIDITY	NKCM	47	85.5
	HCV	4	7.3
	AIHA	2	3.6
	Pure White Cell Aplasia	1	1.8
	Gaucher Disease	1	1.8
SERUM FERRITIN (ng/mL)	24-336	4	7.3
	337-1000	7	12.7
	>1000	44	80
CONSANGUINEOUS MARRIAGE	Yes	43	78.2
	No	12	21.8
SGPT (U/L)	SGPT High	12	21.8
	SGPT Normal	43	78.2
VIRAL MARKERS (HBV & HCV)	HCV	4	7.3
	Non Reactive (both)	51	92.7
DCT	Positive	3	
	Negative	14	

**Figure 1:**
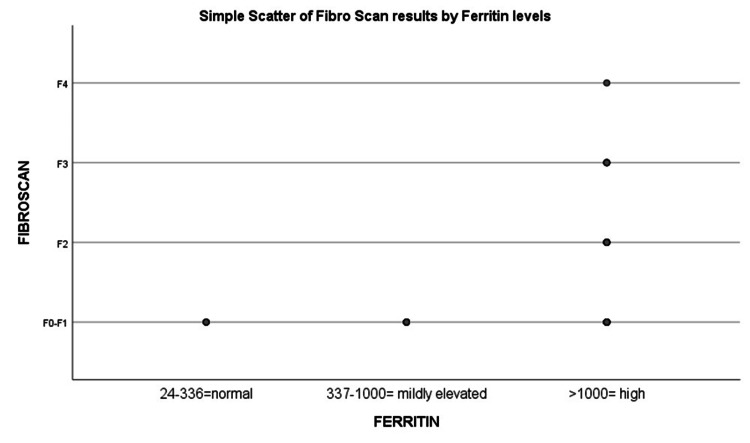
Correlation figure between Fibroscan and Ferritin levels

Spearman's correlation coefficient (r) measures the degree and direction of the relationship between the two variables. Our study's Spearman correlation coefficient was 0.287, indicating a moderately positive relationship between serum ferritin levels and hepatic fibrosis. The p-value of 0.033 is below the significance threshold of 0.05, which indicates that serum ferritin levels and hepatic fibrosis correlate statistically (Figure 1 and Table 3).

**Table 3:** Spearman’s correlation analysis

Correlations				
			FERRITIN	FIBROSCAN
Spearman's rho	FERRITIN	Correlation Coefficient	1.000	0.287^*^
		Sig.	.	0.033
	FIBROSIS	Correlation Coefficient	0.287^*^	1.000
		Sig.	0.033	.

## Discussion

This is the first study done in Pakistan that investigated the correlation between serum ferritin levels and hepatic fibrosis, as confirmed by Fibroscan, in patients with beta-thalassemia. Consistent with previous research, our findings demonstrated a statistically significant positive correlation (r= 0.287; p= 0.033) between serum ferritin levels and hepatic fibrosis. In a study by Mohamed et al. [11], serum ferritin levels were substantially correlated with hepatic fibrosis in thalassemia major patients. Several other studies [12-17] have also reported a significant positive correlation between serum ferritin levels and hepatic fibrosis. The correlation between serum ferritin levels and hepatic fibrosis in beta-thalassemia patients is supported by the findings of our study and the studies cited previously.

In other words, it is unlikely that the observed correlation occurred by coincidence, and there is evidence to support the notion that serum ferritin levels and hepatic fibrosis are correlated. However, it is essential to note that correlation does not always imply causation, and additional research is required to establish causality between the two variables.

In this study 72.7% of the patients were male and 27.3% were females which is consistent with another study [18]. The majority of patients (94.5%) had beta-thalassemia major, consistent with findings by Aydinok et al. [19].

The mean age of the patients was found to be 7.9 years, consistent with the study by Yazal Erdem et al. [20]. In contrast, in a study conducted by Sadullah et al. [21] in Iraq, the mean age of beta-thalassemia patients was found to be 13 years, which is higher than the mean age in our study (7.94 years). Alvi et al. [22] reported that the mean age at diagnosis of the thalassemic patients was seven months, comparable to our study (9.45 months). A study conducted in Pakistan [23] found that the mean age at first transfusion was 1.95 years, which is higher than our study (8.48 months). The study also found that the transfusion frequency varied depending on the type of thalassemia, with beta-thalassemia patients requiring transfusions every two to eight weeks, similar to the transfusion interval reported by Yilmaz et al. [24].

This study found that the majority of patients (80%) had high serum ferritin levels (>1000 ug/mL), followed by mildly elevated levels of serum ferritin (12.7%), and finally, normal levels of serum ferritin (5.5%), which is consistent with the results reported by Fraquelli et al. [25]. In terms of fibrosis stage, most patients (74.5%) had F0-F1 stage fibrosis, consistent with the previous study [25]. While Poustchi et al. [26] reported contrasting results where the majority of the patients had F2 and F3 stage fibrosis.

Most patients (85.5%) had no comorbidities besides beta-thalassemia. Among the comorbidities found, the high prevalence of HCV (7.3%) among these patients was consistent with previous studies in Pakistan [27,28]. The second most prevalent comorbidity was autoimmune hemolytic anemia (AIHA) at 3.6%. The study also found that 78.2% of the patients had parents who were first cousins, consistent with the high prevalence of consanguinity in Pakistan reported by other studies [29,30].

The median value of SGPT was 38 IU/L, with a minimum value of 9 IU/L and a maximum value of 314 IU/L, which is higher than the values reported by Ferraioli et al. [16] at 27 IU/L. The mean creatinine levels for our study were found to be 0.8 mg/dl, which is comparable to the results of previous research [11].

Overall, the results of these studies suggest that there may be variation in the variables of interest among beta-thalassemia patients across different populations. These differences in results could be attributed to patient population variations and the methods used for data collection and analysis. However, further research is needed to confirm these findings and determine the factors contributing to these variations.

There are some limitations to this study that need to be considered. First, the study was conducted at a single-center with a small sample size, limiting the findings' generalizability to other centers and populations. The study did not collect data on potentially important variables, such as monitoring frequency of ferritin levels, iron chelation, family income, education level, and lifestyle factors, which could have affected the outcomes. Furthermore, this study did not assess the long-term outcomes of beta-thalassemia major, which could be important for determining the effectiveness of treatments and interventions. Finally, the study relied on self-report for some variables, which could introduce measurement bias.

## Conclusions

The current study examined the correlation between serum ferritin levels and Fibroscan detected hepatic fibrosis in patients with thalassemia major. According to the findings, the levels of serum ferritin levels in beta-thalassemia patients can be a potential predictive factor in assessing hepatic fibrosis in such patients. This study's findings also have important implications for clinical practice, particularly for the early diagnosis and treatment of hepatic fibrosis in this patient population and regular monitoring of ferritin levels. With a significant difference, clinicians can employ the use of serum ferritin as a surrogate for hepatic fibrosis to minimize the financial burden on patients.
